# 22 Comprehensive Review in the Development of an Occupational Therapy Burn Fellowship Program

**DOI:** 10.1093/jbcr/iraf019.022

**Published:** 2025-04-01

**Authors:** Heather Dodd, Hayley Mata-Whitmer

**Affiliations:** University of North Carolina Jaycee Burn Center; University of North Carolina Jaycee Burn Center

## Abstract

**Introduction:**

The American Burn Association (ABA) advocates for burn surgical fellowships to develop skilled practitioners in burn care. Through hands-on training, fellows are prepared to enhance patient outcomes in burn surgery. Occupational Therapists (OTs) play a vital role in helping survivors regain function, yet opportunities for specialized training are limited. Our accredited OT Burn Fellowship (OTBF) program, supported by the American Occupational Therapy Association (AOTA), has successfully trained eight OTs, empowering them to deliver quality care that aligns with ABA’s competencies for Burn Therapist (BT-C) Specialty Certification.

**Methods:**

The OTBF is a 12-month position with a competitive salary and benefits. Fellows provide patient care while participating in didactic education, mentored clinics, and research.

The curriculum consists of focused learning modules on burn care, including basic medical principles, ethics, intensive care, upper extremity management, neck/facial treatment, inpatient/outpatient rehabilitation, and research methodologies. Throughout, data is collected to evaluate various outcomes, including knowledge checks, contribution of continuing education units (CEUs) for peers, productivity benchmarks, mentor accrued hours and feedback, and overall program evaluation through post-fellowship surveys. After concluding the research module, fellows aim to have their studies accepted for presentation at a burn conference or publication in a peer-reviewed journal.

**Results:**

Upon completing the fellowship program, all fellows achieved a minimum of 80% on module assessments and delivered at least seven hours of CEUs focused on evidence-based research. They also provided six hours of academic instruction and hands-on tutorials to a local OT program. All fellows completed their research and presented at a burn conference. They met or exceeded 85% of productivity standards for direct patient care, contributing to hospital revenue. Throughout the year, they accrued over 350 mentored hours with experienced therapists and interdisciplinary team members.

Fellows fulfilled AOTA and ABA competencies for fast-track candidacy for Board Certification in Rehabilitation and ability to apply for BT-C upon completing the required hours. As a result of our nationally accredited OTBF program, 75% of our fellows are now employed in burn centers, providing exceptional rehabilitation across the country.

**Conclusions:**

The OTBF has proven beneficial within the hospital system and in burn care. We are dedicated to continually evaluating our program to enhance therapist quality by assessing educational opportunities and implementing changes aligned with current practices. We see potential in promoting OT fellowship programs in other centers to increase the number of competent burn therapists.

**Applicability of Research to Practice:**

The OTBF plays a crucial role in developing skilled therapists and advancing research in burn care.

**Funding for the Study:**

N/A

